# On the Anatomy of Health-related Actions for Which People Could Reasonably be Held Responsible: A Framework

**DOI:** 10.1093/jmp/jhad025

**Published:** 2023-05-31

**Authors:** Kristine Bærøe, Andreas Albertsen, Cornelius Cappelen

**Affiliations:** University of Bergen, Bergen, Norway; Aarhus University, Aarhus, Denmark; University of Bergen, Bergen, Norway

**Keywords:** priority setting, responsibility in health, risk-sharing, risky choice, unhealthy lifestyles

## Abstract

Should we let personal responsibility for health-related behavior influence the allocation of healthcare resources? In this paper, we clarify what it means to be responsible for an action. We rely on a crucial conceptual distinction between being responsible and holding someone responsible, and show that even though we might be considered responsible and blameworthy for our health-related actions, there could still be well-justified reasons for not considering it reasonable to hold us responsible by giving us lower priority. We transform these philosophical considerations into analytical use first by assessing the general features of health-related actions and the corresponding healthcare needs. Then, we identify clusters of structural features that even adversely affected people cannot reasonably deny constitute actions for which they should be held responsible. We summarize the results in an analytical framework that can be used by decision-makers when considering personal responsibility for health as a criterion for setting priorities.

## I. INTRODUCTION

The latest study on the global burden of disease confirms that non-communicable diseases are increasingly prevalent (GBD Causes of Death Collaborators, 2017). Our choices concerning nutrition, exercise, alcohol, and smoking influence the prevalence of such diseases, which include heart disease, stroke, and type 2 diabetes. We define “unhealthy lifestyles” as “continuous actions that have been shown to have an adverse effect on individuals’ health.” It can sometimes be difficult to assess objectively whether any particular lifestyle is unhealthy (e.g., one that includes excessive exercise), but less controversial examples of unhealthy habits include smoking, excessive drinking, eating sugary and fatty food, and little, or no exercise. Furthermore, diseases and injuries often occur in relation to choices we might make on a less regular basis, such as participating in hazardous sports, having unprotected sex, or driving a motorcycle without a helmet. Those actions that pose a threat to one’s health but are not carried out regularly are referred to here as making a “risky choice,” rather than the stronger claim that they are necessarily indicative of an “unhealthy lifestyle.” Nevertheless, in both cases, a subsequent need for health care gives rise to discussions about personal responsibility as a rationing criterion for allocating scarce healthcare resources. In practice, this concern translates into questions about whether we should tax risky behavior (including lifestyles or particular actions) or give lower priority at the point of care to those deemed responsible for their treatment needs. The supposed fairness of such solutions has of course been widely debated (Reiser, 1985; Cappelen and Norheim, 2005; Buyx, 2008; Feiring, 2008; Schmidt, 2008, 2009; Nielsen and Axelsen, 2012; Andersen, 2013, 2014; Brown, 2013; Albertsen, 2015; Albertsen and Knight, 2015; Bærøe and Cappelen, 2015; Andersen and Nielsen, 2016; Friesen, 2016; Brown, Maslen, and Savulescu, 2019; Levy, 2019; Traina, Martinussen, and Feiring, 2019).

The issue is complicated for several reasons. First, it is philosophically challenging to conceptualize exactly what it means to be responsible. Second, there are different ways to hold people responsible in terms of the kind of sanction that is implemented. Third, there are practical difficulties involved in assessing responsibility and implementing sanctions. On top of this, conceptualizations of responsibility, and coherent strategies for implementation need to be adequately contextualized in a healthcare setting. This means that how, and under what conditions, people should be held personally responsible for their health conditions, and associated treatment needs is an issue that should not be discussed in a vacuum; instead, it must be considered in light of other moral values that are structuring the healthcare system, and the objectives of the care provided. Thus, even though we would have theoretically justified reasons for considering people responsible for their own health, other principles may override our reasons to *hold* them responsible in terms of introducing sanctions. Thus, strong reasons to consider people responsible for their own health are necessary but not sufficient to justify *holding* them responsible.

The main objective of this paper is to advance the discussion of personal responsibility in the allocation of healthcare resources. We do not aim to further develop the philosophical discussion on responsibility as such. The novelty in our approach is to be found in the translational move we facilitate between established conceptualizations of responsibility, normative argumentation, and an analytical tool for real-world policy-making. This paper addresses a real-world practical challenge when asking whether it is acceptable to use personal responsibility for unhealthy lifestyles or risky actions as a criterion for healthcare distribution. Our response rests on the assumption that an adequate answer needs to take certain aspects of the real-world, such as feasibility and contextualization, into account. In the following, we start by (1) clarifying how the focus of this paper is delimited, and (2) presenting the innovative architecture of the argumentative reasoning used here, which represents the “translational move” we introduce to navigate between theoretical considerations of responsibility and the call within practice for justified and fair policies. We use certain insights from philosophy to identify anatomical structures of the kinds of health-related actions for which it could be fair to hold people responsible. An overview of these structures, or particular clusters thereof, can be used by decision-makers when assessing whether a particular kind of “lifestyle/risky choice needing health care” pairing should be allocated resources. To get there, our next step is (3) to demonstrate how a rather uncontroversial conceptualization of “moral responsibility” based on ideal, decontextualized reasoning can be seen as relevant to a conceptualization of responsibility for health-related actions. In this part of the discussion, we argue that the specified conditions of “control,” “quality of will,” and “knowledge” can be used to assess the acceptability of holding people responsible for their health-related actions and to establish a set of conditions indicating what it takes to be *responsible*. We then contextualize these ideal perspectives on responsibility by (4) discussing how decision-makers cannot hold people responsible in terms of sanctioning policies that clearly undermine essential values within healthcare provisions (here identified according to the four general principles of bioethics). Finally, we (5) identify a cluster of structural features of actions and health outcomes specifying when and how responsibility could reasonably be accounted for in healthcare distribution. In doing so, we will have translated the theoretical debate into a framework for practical usage; the resulting set of generic criteria of actions and conditions can be a helpful analytical tool for decision-makers assessing substantive policy proposals.

## II. CLARIFICATIONS

“Holding people responsible” is a term with many different meanings, particularly in a healthcare setting. Here, the notion encompasses any decision that sanctions people due to their exercise of responsibility. For instance, one way to hold a patient responsible for her treatment needs is to give her lower priority on the waiting list (Veatch, 1980; Dworkin, 1981). Perhaps the strictest responsibility mechanism is to deny a patient treatment altogether if she is responsible for her own illness. Within the context of a publicly funded healthcare system, this way of holding people responsible is defended by Rakowski (1993). A more lenient mechanism would be to give lower priority to treating diseases that are related to unhealthy lifestyle choices. To illustrate, a patient who has alcohol-related end-stage liver disease receives lower priority on the waiting list than one whose need for a liver transplant cannot be traced to lifestyle (Thornton, 2009; Albertsen, 2016). Increased co-payments is yet another measure (Andersen, 2014), as are higher premiums levied on health insurance for customers with unhealthy lifestyles (Veatch and Steinfels, 1974). Risky choices could also be taxed to compensate for the aggregated increase in associated treatment costs (Cappelen and Norheim, 2005, 2006).^1^

We can have different reasons to hold people responsible for their behavior when distributing scarce healthcare resources. Two such reasons have been particularly prominent (Cappelen and Norheim, 2005). First, one could consider it *fair* that those who are responsible for their health disadvantages are given lower priority. Such arguments often emerge from the responsibility-sensitive view on distributive justice known as luck egalitarianism (Segall, 2010; Le Grand, 2013; Albertsen and Knight, 2015; Albertsen, 2020). Second, *efficiency* concerns also matter, both because the total cost of health care depends on how risky people’s choices are, and because policy measures targeting risky behavior might disincentivize such behavior. Fairness and efficiency considerations differ in important ways. According to the latter, holding patients responsible may have a positive effect on their future behavior and thus decrease the total amount of resources to be spent on their health care. According to the former, if people are free to make choices, then we are morally justified in holding them responsible for the consequences (independent of whether this affects future behavior). In this paper, we focus on whether fairness considerations can support responsibility catering policies; we leave efficiency considerations mostly aside.^2^

### The Architecture of Our Line of Reasoning

In the literature on priority setting in health, the need for ensuring the *legitimacy* of the practical conclusions has been broadly endorsed (Kapiriri, Norheim, and Martin, 2007; Bærøe, 2008; Maluka et al., 2010; Bærøe and Baltussen, 2014). The idea behind the conceptualization of “legitimacy” is that even though we cannot expect people to agree on substantive distributional principles, they can be expected to agree on a procedure to arrive at such conclusions (Daniels and Sabin, 2002). It has been argued that policy-makers must make particularly strong efforts to ensure that the rationales for setting limits to certain healthcare services are well-justified and available to those who are adversely affected by the decision (Bærøe and Baltussen, 2014). This is to ensure that the proper *moral authority* has been exercised to make these decisions. How should we take this general idea into account when discussing personal responsibility as a distribution criterion? Our approach here is first to identify who the adversely affected are, followed by a presentation of the argumentative structure of how we can filter out various generic features of actions for which it is *not reasonable* to hold people responsible. We thereby end up with reasons for holding someone responsible for health that the adversely affected cannot *reasonably* reject.

We define “reasonableness” according to Rawls’ conceptualization of reasonable persons as ones who are rational in the sense that they deliberate over their own ends, interests, and life-plans, as well as their priorities and how to achieve them. However, such an individual is also characteristically moved to “. . . desire for its own sake a social world in which they, as free and equal, can cooperate with others on terms all can accept” (Rawls, 1993, 50). Reasonable people “. . . are ready to propose principles and standards as fair terms of cooperation and to abide by them willingly, given the assurance that others will likewise do so. Those norms they view as reasonable for everyone to accept and therefore as justifiable to them; and they are ready to discuss the fair terms that others propose” (Rawls, 1993, 49). We use the adjective “reasonably” to indicate the presence of the structural constraint reasonable persons put on their assessments, that is, the aim of promoting terms that all can accept. “Unjustified reasons” refers to reasons for an action or a policy that cannot be accounted for accordingly.

### Adversely Affected

The adversely affected by responsibility ascription would obviously be those who, for example, must pay higher taxes/premiums, and those who are given lower priority at the point of care. In addition, the next of kin to those who are held responsible might also be adversely affected by the practical and emotional consequences of extra expenses or rationed health care. Because of the responsibility ascription related to increased premiums/out-of-pocket expenditures, some might drop out of testing, treatment, and follow-ups due to a lack of money or other issues that can make it difficult for them to take on the burden of responsibility. For those with a contagious condition (e.g., positive tuberculosis status), failing to seek testing, and appropriate treatment will adversely affect others who may then unwillingly acquire the condition. Therefore, well-justified rationales for holding someone responsible must address not only those who are directly affected by the decisions, but also those who could unfairly end up being subjected to extra expenses, and the risk that unknown medical status and untreated conditions entail.

### Well-justified Rationales for Policy Decisions About Responsibility

The litmus test of the reasonableness of rationales for policy decisions focusing on individual responsibility is whether the adversely affected may have strong counterarguments. Thus, our overall argumentative strategy is to look for rationales to which the adversely affected cannot reasonably object. We approach these by filtering out responsibility-related features of actions and circumstances that are well-justified reasons for which one should not be held responsible. Then, we can assume (until proven otherwise) that the remaining responsibility-related features constitute actions for which one can reasonably be held responsible.

Two different sets of rationales can be filtered through this litmus test. First, when assessing whether people are considered responsible for their actions, decision-makers cannot apply a conceptualization of responsibility that reflects those rationales that the adversely affected have good reasons to find unreasonable. This part of the filtering process addresses both the responsibility for actions assessed at a point in time when the need for health care is only a future possibility, and the responsibility assessed at the time when people need health care. As we will see, in the latter situation, more information is required to be able to reasonably hold people responsible for their healthcare needs. Second, to justify sanctions (when the scope of responsibility for a health-related action is established), the decision-makers must take into account how the suggested policies might conflict with other well-established values that structure the healthcare sector. Decisions involving conflicting values can only be justified as legitimate if they do not lead to circumstances to which the adversely affected can reasonably object (including circumstances to which any user of the healthcare system would have reason to object).

Thus, we propose a two-step argumentative approach:

I. We identify a conceptualization of responsibility in health to which the adversely affected cannot reasonably object, which we subsequently use to filter out unjustifiable reasons both regarding assessment at the point of time when the action takes place, and when the healthcare need occurs.
II. We discuss how the values that are reflected in the four principles of biomedical ethics and associated with the ethos of health care or the organization of healthcare systems (at least in a large part of the Western world) may conflict with responsibility policies. When responsibility catering policies undermine the inherent and valued structures of the healthcare system itself, it would not be reasonable from the perspective of the adversely affected (or others, for that matter) to introduce such policies. At this point, we are then left with a set of risky and lifestyle-related actions for which it could be reasonable to hold people responsible.

## III. FIRST FILTRATION: CONDITIONS FOR CONSIDERING SOMEONE RESPONSIBLE FOR HEALTH-RELATED ACTIONS

In this section, we investigate the general conditions that must be met for someone to be responsible for health-related behavior and for actions actually materializing into healthcare needs. The relevance of this distinction has to do with the practical measures that are available for holding people responsible—they can be implemented at the point when a call for healthcare treatment is only a future possibility, or when people actually need care.

### Moral Responsibility and Responsibility for Health-related Actions

In the philosophical literature on moral responsibility, being responsible for a morally objectionable action is typically identified with being blameworthy.^3^^,^^4^ While we employ the notions of blameworthiness and wrongdoing used in the philosophical and legal literature on responsibility in health, we do not make the initial assumption that people are committing a moral wrongdoing when they make risky choices related to their health. They might do so, of course, but this is beside the point of our discussion. Since we do not presuppose that health-related actions are morally wrong, we are not claiming that people responsible for such risky choices deserve moral blame. However, it seems plausible that the conditions necessary for making an agent responsible for risky choices are relevantly similar to the conditions necessary for making agents blameworthy for morally wrong actions. The conditions we identify below seem relevant even in non-moral cases.

The fact that an agent is responsible for an action does not always justify holding her responsible for that action. If an agent is blameworthy for X, it is appropriate to blame her.^5^ But, the fact that blame is appropriate only provides *pro tanto* reasons for blaming that agent (McKenna, 2012). The reasons any particular agent has for actually expressing this blame or holding the blameworthy agent responsible can easily be outweighed. Sometimes blaming someone leads to disastrous outcomes. For instance, if a person is deeply depressed, you will have very good reasons not to blame her, even though she is blameworthy.^6^ In the case of assessing responsibility for health-related actions, and holding people responsible, contextual factors may impact their blameworthiness.

It is common to distinguish three different conditions that need to be satisfied for an agent to be blameworthy for a wrongful/objectionable/morally wrong action. First, many philosophers claim that there is a *quality of will* condition on moral blameworthiness (Strawson, 2005; McKenna, 2012; Shoemaker, 2015). To be blameworthy for an action or omission, the agent must display a lack of proper regard for others. Sometimes agents act with ill will, but more often they act with an insufficiently good will. We typically experience reactive attitudes when we encounter malign intentions or a lack of proper concern for others. The point of an excuse is often to show that one’s action, contrary to appearances, did not express any lack of proper regard (Levy, 2005). At the very least, the quality of will with which one acts typically influences his/her degree of blameworthiness. A relevant example from the healthcare sector could be base jumpers getting hurt in impassable terrain and thereby putting the life of their rescuers at risk, as well as occupying resources from which others could have benefited. Another example could be healthcare personnel who do not take sufficient measures to protect themselves from epidemics while working with vulnerable patients who are prevented from doing the same. On the one hand, we might be inclined not to judge actions as blameworthy when, for example, health personnel knowingly expose themselves to contagion when lacking available protection so as to care for sick people who would otherwise have been left on their own. On the other hand, we are probably more inclined to consider those who are seeking hazards for the sake of adrenaline kicks with some disapproval. Similarly, we might also be inclined to tolerate higher risks if the risk-taking is connected to a means to realize a necessary objective. Thus, the quality of will may have a function in the assessment of “moral responsibility,” which is then reflected in people’s more intuitive judgments of health-related actions. However, the quality of will as such will not play any relevant role in conceptualizing the responsibility for health-related actions, since these often will not even involve others at all. For that reason, we leave aside the discussion of the impact of the quality of will on the responsibility for health-related actions here.^7^

The *control condition* on moral responsibility concerns whether an action (omission) is *up to us* in the relevant sense. It is common to distinguish between *direct* and *indirect* control (Zimmerman, 2008; Portmore, 2019).^8^ Let us consider a case of drunk driving. Sam drives home from a party where he has had too much to drink. In his intoxicated state, he loses control of his car, and kills a pedestrian. Sam did not have direct control over the quality of his driving, but he appears to be blameworthy for the death of the pedestrian. A plausible explanation of why this is the case is because Sam was in control of his decision to drink at the party. In virtue of this decision, he has *indirect* control of the quality of his driving. Another common distinction is between *volitional* control and *rational* control. Volitional control is the kind of control we exert over our intentional actions (Levy, 2014; McHugh, 2017; Portmore, 2019). To have volitional control over an action means that your action is governed by your will: you can perform it by deciding, trying, or intending. Whether to have a sip of water or scratch your nose is under one’s volitional control, but whether to believe that 2 + 3=5, or to feel elated, or melancholic is typically not.^9^ Nevertheless, philosophers often maintain that there is a different sense in which beliefs, emotions, and other reasons-responsive attitudes are under our control. We can be said to have *rational control* over such attitudes, insofar as they are connected in the right way to our underlying evaluative judgments (Smith, 2005; McKenna, 2012; McHugh, 2017; Portmore, 2019).^10^ We exercise rational control over our reasons-responsive attitudes by forming, sustaining, revising, or abandoning these attitudes in light of facts that we take to count for, or against them. Although these attitudes cannot typically be changed at will, they reflect our evaluative judgments in a way that recalcitrant emotions, sudden urges, and irrational beliefs do not.

What kind of control is necessary in order to be blameworthy? This is a highly contested issue, which we do not attempt to settle in this paper.^11^ One reasonable approach to addressing the issue is by asking whether the agent had a *fair opportunity* to avoid the wrongdoing (Brink and Nelkin, 2013). It is uncontroversial to claim that the control condition can be violated by states such as compulsions, paralyzing fears, irresistible desires, or clinical depression, as well as external forces, as in the case of coercion, hypnosis, or brainwashing. Moreover, agents lack the relevant control if they do not have the opportunity to do what they ought to do (assuming they are not responsible for the lack of opportunity), or if it is extremely difficult to do what they ought to do.^12^ To illustrate, if stores do not provide vegetables, and one cannot grow vegetables at home, people cannot be held responsible for not eating vegetables. Logically, there must be healthy options available for people to be held responsible for choosing the less-healthy alternatives. Furthermore, even if vegetables are available in stores, people must be able to reach the stores and pay for them. In other words, even in the presence of alternatives, people cannot be held responsible unless they can feasibly benefit from these alternatives. Building on this, other factors may also limit the extent to which the control requirement is satisfied in relation to people’s health-related choices. Social and cultural influences may constitute barriers to healthy lifestyle choices. For instance, estimates indicate that 20 percent of the adult workforce in the United States smokes (Heidenreich et al., 2011). Closer examination of the numbers reveals that the percentage of smokers increases drastically among low-income and low-educated workers (Heidenreich et al., 2011). This indicates that socioeconomic circumstances correlate with smoking. More generally, health-related choices are often influenced by factors beyond personal control, such as socioeconomic status, socialization, and family influence.

The third condition is the *epistemic condition.* It aims at specifying the amount of knowledge and awareness that is required to be blameworthy for an action or omission. For an agent to be blameworthy for an action or omission, she needs to be a morally responsible agent. This requires the capacity to recognize moral wrongdoing and moral reasons. Small children are often exempted from moral responsibility because they lack the general capacity to recognize moral reasons. However, a lack of knowledge or awareness can also be an excusing factor for agents who *possess* this general capacity. We can therefore distinguish between different kinds of intentional states relevant to wrongdoing. An action is *purposeful* if the agent deliberatively did something she knew was morally wrong. While this is relevant in a moral discourse, we do not recognize its relevance for health-related actions. An action is *reckless* if the agent is aware that the action poses an unjustifiable risk. When translated into a health-related setting, we could say that a person deliberatively did something she knew involved a high risk of getting ill or injured. An action is *negligent* if she is unaware that her action poses an unjustifiable risk, although she should have known. Finally, there are risky actions in which the agent neither knew nor should have known about the risk they posed. Such actions are blameless. Both *purposeful* and *reckless* wrongdoing are commonly taken to be blameworthy. In both cases, an agent is in control of her action and displays an insufficient concern for the unjustified risk involved in the case of recklessness, and an ill will in the case of purposefulness. Negligence, along with unwitting omissions—as when one forgets one’s dog in a hot car—poses a difficult problem for theories of responsibility, because the wrongdoing does not directly result from any *choice* to act wrongly: the dog-owner did not choose to forget his dog in the car (Moore and Hurd, 2011; Clarke, 2014). There are two main competing strategies for explaining how negligence and unwitting omissions nevertheless may be blameworthy. Some maintain that choice is a necessary condition for being blameworthy. This means that the wrongdoing must be traced back to a previous conscious choice (e.g., not checking the brakes on the bicycle) (Rosen, 2004; Zimmerman, 2008). In such cases, negligent agents may still be blameworthy for their actions because their ignorance itself is culpable.^13^ Others maintain that no prior choice is necessary for blameworthiness. The negligent action or omission itself may often reflect an insufficient concern for others, which itself may be blameworthy (Smith, 2005; Sher, 2009).

Many aspects of the epistemic condition and the above discussion are clearly relevant for health-related choices, including, for example, available information. The amount of processed information about the potential consequences of a lifestyle act required to make autonomous actions is an important consideration. A condition of being fully informed, as is required for consenting to participate in medical research, seems too strict, as this would not be the condition on which “normal choosers” act in their daily lives. However, if someone is completely ignorant of health-related consequences due to a lack of available and relevant research, she will not be responsible. For example, the first smokers in the world were not responsible for unhealthy risk-taking, because serious consequences were not yet known.^14^ People’s levels of understanding cannot be assessed in isolation from the contextual circumstances. In fact, at least three features are relevant to consider in combination: the extent to which the knowledge is established, the extent to which the knowledge is available, and the actual risk involved.

### Conditions for Being Responsible for Health-related Actions

Based on the conceptualization of moral responsibility, we can now summarize different conditions that will have to be present when considering someone as responsible for health-related choices. Since being responsible is a necessary condition for appropriately holding someone responsible (but not sufficient), these conditions will have to be present when someone is held responsible for their actions in terms of sanctions, too.

At this point, it is important yet again to emphasize the distinction between sanctions being introduced before there is a need for health care, and sanctions being introduced when the need is present. Table 1 provides a preliminary overview of the generic conditions that allow us to consider the extent to which a person is responsible for a health-related action. Thus, these conditions are relevant to take into account when decision-makers consider introducing “risk-sharing” sanctions for specific health-related actions (in terms of, e.g., increased premiums, and taxes).

**Table 1.** An overview of general conditions that need to be satisfied for an agent to be responsible for a health-related action

Control conditions	Epistemic conditions
■ Sufficiently free of controlling influences (e.g., psychological states that undermine one’s ability to respond to reasons) ■ Alternative(s) involving less risk is/are available ■ Benefiting from less-risky alternatives is feasible	■ Capacity to understand “wrongdoing”/risk, including: (1) Negligence (acting in a way that poses a risk, although one should have been aware of it) (2) Recklessness (knowingly ignoring the action poses an unjustifiable risk)

### Conditions for Being Responsible for Needs for Health Care

When assessing whether it is reasonable to hold people responsible *when they need care* (i.e., at the bedside), the previous conceptualization of the responsibility for actions is not sufficient to state that they are responsible for such a need. If policy-makers wish to go beyond risk-sharing measures and introduce sanctions “at the bedside,” further criteria have to be accounted for in order for responsibility catering policies to be fair. To go from considering a person responsible for an action to considering a person responsible for an outcome of an action (or a series of choices/actions) involves an extra layer of complexity; we have to know more about the circumstances of the relevant action(s) and the connection to the outcome before we could be justified in giving lower priority to a person based on responsibility considered at the bedside.

First, when considering individual responsibility *ex post* at the bedside, we would need to know retrospectively whether there is a *causal connection* between a patient’s previous behavior, and her need for treatment. If there is no such connection but her care is nevertheless being adversely affected by a sanction, she is held responsible for too much. Those being held responsible with a sanction at the bedside are being treated differently from people with the same conditions who did not have the same lifestyle or took a risky choice. To justify unequal treatment of equal cases, there must be a relevant difference between them. If one cannot establish with a high level of certainty that unhealthy behavior or a risky choice caused the conditions for one and not the other, the unhealthy action cannot justify different treatment. In the case of higher taxes/premiums, people with risky lifestyles are collectively held responsible for the aggregate increase in treatment costs they impose on the healthcare budget ex ante. At that point, causality between behavior and potential healthcare needs in *individual* cases is not relevant; the individual’s risk-taking is.

Further specifications of the control/epistemic conditions for moral responsibility also seem to be required. To be responsible for contracting a healthcare need, one must be aware of both the threat of being exposed to damage or illness as well as being in control of avoiding the exposure. The latter means responsibility for the healthcare need implies that contracting the condition could have been avoided by actions of protection (e.g., getting vaccinations before traveling in tropical areas, or using helmets, or condoms), and that measures to do so are available. Moreover, it seems to make a difference whether people are deliberatively seeking situations that expose them to illness or damage, or whether one is passively, or even unwillingly, encountering these situations in pursuing something else (like caring for contagious patients). If these conditions of available protection, awareness, and voluntary exposure are not satisfied, we are not justified in accepting responsibility as a criterion for giving some kind of lower priority at the bedside. How more precisely to conceptualize/operationalize “available protection,” “awareness,” and “voluntariness” is not a straightforward matter, and it is also beyond the scope of this article. Nevertheless, we are required to enable assessments of actions where these criteria are present. We assume the burden of proof lies with those who are assessing and not those in need of health care, and make some tentative suggestions about the necessary, but not necessarily sufficient, conditions. “Available protection” implies that the protective effect of devices is common knowledge, and that such devices are reachable without requiring a lot of resources. We claim that “awareness” cannot be assumed unless the threat involved is common knowledge, and “voluntariness” can only be ascribed in cases where there is a lack of any identified or patient self-reported influences (broadly construed to include the moral intentions of pursuing the good of others).

Table 2 presents an overview of these further factors that, we have argued, should be considered when contemplating sanctions based on responsibility at the bedside. These factors are relevant since their presence either (1) undermines or supports any relation between the action and the condition, (2) influences the control condition, or (3) influences the epistemic condition.

**Table 2.** Additional features to consider when introducing responsibility-based policies “at the bedside” that allow for treating people with equal conditions unequally

Relation between action and condition	Ways of catching the condition	Ways of avoiding the condition
■ Traceable causality ■ Untraceable association	■ Exposure requires active effort ■ Exposure can happen without any effort ■ Exposure can happen either with or without any effort	■ There are no ways to avoid the condition. ■ There are ways to actively protect oneself from the condition ■ There are ways to completely avoid the condition

### Structural Features of Actions: First Filtering

We now use the discussion above (summarized in tables 1 and 2) in a first step to filter out features of actions for which one can reasonably be considered responsible. As the consequences of both risk-sharing policies and receiving lower priority can be quite severe, the adversely affected have robust reasons for requiring clearly restricted interpretations of the conditions for being considered responsible, which translates into strict requirements involving low probability for being wrongly assessed as responsible. This means that the decision-makers will not have the moral authority to hold someone responsible, if they do not develop the proposed policies accordingly. Structurally, applying the criteria of responsibility can be presented as an exemption from the otherwise default standard of not putting weight on responsibility, and only in cases where there is a minimum risk of wrongly ascribing responsibility. Later in this paper, we say more about the generic anatomy of such cases.

While imagining the perspective of the adversely affected, we can summarize the preliminary content of these requirements. First, the adversely affected cannot accept the ascribed responsibility if they are acting under a lack of control in terms of strong influences. Moreover, one cannot accept being treated as responsible if one is (innocently) completely ignorant of the risk (e.g., exposed to contagion by someone without symptoms), or has no feasible alternatives to risky behavior. Second, when having contracted a condition, or disease, blameworthiness must include the additional condition that there is a well-established causal relationship in medical terms between previous actions, and the healthcare need. Furthermore, in cases of having a contagious condition, blameworthiness must involve the conditions that (1) one was familiar with the ways of being exposed to and avoiding the harms in question at a point, and (2) one must have been capable of making adequate risk assessments (leaving out cases of becoming sick while sleeping or being unconscious).

## IV. SECOND FILTRATION: CONDITIONS FOR HOLDING SOMEONE RESPONSIBLE FOR HEALTH-RELATED ACTIONS

While the factors listed thus far, as suitably specified, provide us with the tools to assess whether people *are responsible* for their health-related actions and their subsequent diseases/conditions, we argue that they are still insufficient to determine how, and to what extent we should *hold* them responsible in the allocation of healthcare resources. Reasons for holding someone responsible do not occur in a vacuum. Rather, the circumstances within which an individual can be deemed responsible for his or her health-related behavior are at the same time embedded in broader contexts of social and cultural values, including those that are shaping the national healthcare systems. Thus, an overall justified distribution of fair health care must consider to what extent holding someone responsible conflicts with other surrounding values, and whether the latter should override the first. It is open to discussion exactly where to draw the boundaries for the relevant context. However, as criteria for deciding on individual entitlements to care at the same time constitute parts of the healthcare system, considering the more fundamental values of this system seems a defensible place to draw the line.

In the following, we submit that several additional factors affect the appropriateness of holding people responsible for certain measures. These are factors that do not diminish a person’s responsibility for a specific health disadvantage, but rather diminish the appropriateness of introducing responsibility-sensitive measures for allocating healthcare resources. They represent values that members of the society in general—and the adversely affected in particular—have no obviously good reasons to reject. Thus, we are now moving from identifying relevant conditions for being responsible to considering additional reasons for not holding someone responsible, based on societal values and bioethical principles. These values can make it such that the necessary condition of “being responsible” is an insufficient condition for “being held responsible.”

### Risk-sharing Policy: Conflicting Reasons

When assessing the fairness of risk-sharing policies for specific, health-related actions, it is important to avoid unfortunate disincentives, that is, policies that may work against otherwise prudent actions because of increased costs. It would not be reasonable to tax running shoes, for example, because of the increased risk of damage associated with running, given all the well-documented, positive health impacts the use of these shoes could otherwise have. We therefore suggest that fair risk-sharing policies for holding people responsible for health-related actions be limited to actions for which potential positive health effects clearly (as this can be a matter of degree) do not outweigh the potential negative ones. Moreover, to hold people responsible for risk-taking in ways to which the adversely affected cannot reasonably object, there will have to be an undeniable level of risk involved in the action. Also, the more cost-driving the consequences can be expected to be, the fairer a risk-sharing policy may be, as resources are saved for the non-self-inflicted needs of others.

### Sanctions at the Bedside: Conflicting Reasons

We now discuss the option of implementing distributional policies that involve holding people responsible for their health-related actions at the point of care. At this point, such strategies may conflict with the fundamental values on which the healthcare provision is based. The four principles of biomedical ethics are patient autonomy, beneficence, non-maleficence, and justice. (Beauchamp and Childress, 2009). These principles are broadly embraced around the world (especially in the Western world) as central values that serve to protect patients against being controlled or harmed, to promote good quality care, and to ensure that individuals are treated as moral equals when in need of care. They represent core values of the “ethos” that ideally should structure moral reasoning and thereby modern healthcare services. Beauchamp and Childress do not assume prior superiority to any of these principles; they must be balanced against each of the conditions for the situations within which they are applied. We agree on this and take these principles to be so general that they cover a broad scope of more specific principles. The balancing of the principles might differ in diverse cultural contexts. Also, there might be principles like “solidarity” that represent a specific kind of justice, and these might influence the shaping of healthcare institutions in so-called “welfare states” and not in others. Nevertheless, in their unspecified form, they represent prime candidates for relevant, general societal concerns that might override the justification of holding individuals responsible for health-related actions within the healthcare systems. The following discussion of these principles against responsibility policies serves the function of achieving the last step of the filtering process: Even though people are responsible for their health-related actions, fundamental values that constitute healthcare provision may contradict the rationale for also being held responsible for these actions.

### Respecting Patient Autonomy

In the context of health care, respecting patient autonomy refers to the importance of respecting people’s choices, and increasing or maintaining their capacity to make choices (Beauchamp and Childress, 2009). This respect is demonstrated in the requirement of patients’ informed consent; when being fully informed about risks and benefits, patients make their own decisions about receiving health care. Within healthcare services, it is usually assumed that choices are carried out autonomously, unless proven otherwise. This very idea of letting people’s lives fare in accordance with their own autonomous choices lends itself to a discussion of the introduction of responsibility-sensitive policies (Sandman, Gustavsson, and Munthe, 2016). People would be assumed to have acted autonomously when taking a health-related risk, that is, in the sense of having the relevant control and information to be considered responsible. Family, healthcare personnel, and the legal system can contest someone’s capacity for making autonomous choices. Allowing for this aims to protect individuals from being left with a responsibility for their own health when they are not able to attend to it, and from suffering consequences they do not understand. In these cases, benevolence structures the care. On a similar track, benevolence could protect patients from being held responsible for health-related actions that are beyond their capacity to be fully responsible for. But, insofar as people are deemed autonomous by default, it would be left to the patient themselves, not others, to provide the proof of a lack of responsibility if certain choices, and actions were carried out under the strong influence of others or structural mechanisms. The process of proving such evidence would be difficult, if not impossible, and the consequence of this practice would be highly problematic; people who should not be considered responsible for their actions might end up being treated as if they were. Thus, the value of patient autonomy in health care can cut both ways and gives us a great deal of reason for caution.

If we start to question the fundamental value of autonomy when introducing responsibility as a criterion for resource allocation (to avoid inadequate ascription of responsibility), we simultaneously undermine autonomy as a valued, structuring principle of organized health care. Anyone is morally justified to object to such a consequence. At the same time, it is also highly reasonable for those adversely affected by responsibility policies at the point of care to object to the risk of being deemed responsible for something one did not have control over while having poor chances to prove the verdict wrong. Moreover, it is also important to point out that allowing people’s autonomous choices to disadvantage them may negatively affect their capacity for making autonomous choices in the future (Brown, 2005). As we see it, in this situation, the principle of not causing harm, either by holding people responsible for too much or damaging future capacities, outweighs a call to strictly follow the consequences of the principle of promoting patients’ autonomy.

### The Principle of Beneficence

The ordinary meaning of this principle is that healthcare providers should take actions that serve the best interests of their patients; in other words, they should promote the patients’ well-being (Beauchamp and Childress, 2009). Patients should trust that the physician’s chief objective is to help. Clearly, this is a core value of healthcare ethics and widely accepted as the proper goal of medicine. However, the principle of beneficence seems to clash with certain institutional practices of holding patients responsible. To illustrate, consider the rather extreme responsibility policy of denying treatment to a patient who is fully responsible for her illness. Prima facie this seems to contradict the principle of beneficence. If healthcare personnel are expected to differentiate between patients who are entitled to realize their benefits unconditionally and those who are not, this core principle in health care is fundamentally challenged, and trust in the care providers will reasonably come under threat.

An argument in favor of implementing responsibility-based policies also concerns incentives and disincentives. Institutional measures affect people’s behavior. The extent to which we would want to hold people responsible for their health disadvantages would in part depend on how these institutional measures affect how people behave. As mentioned earlier, sometimes we can expect the threat of receiving lower priority to provide people with a further reason to stay healthy. In these cases, there is an additional health benefit flowing from responsibility-sensitive measures. Another argument in favor of responsibility-based policies is that it may very well be that not benefiting those who are responsible for their illness (or benefiting them to a lesser degree) may free up resources and thereby allow the healthcare system to benefit others who are not currently benefiting.

A related but different concern to consider is the extent to which the person in question would be expected to *benefit* from treatment. Denying treatment to a person who would benefit significantly from a treatment inflicts harm that could have been avoided. The more severe the condition is in terms of the loss if not treated and the more benefit if treated, the harsher it seems to hold someone responsible. On the other hand, the propensity to act in specific ways in the future may also curtail the benefit of treatment (Feiring, 2008). In such cases, holding people responsible and prioritizing based on the capacity to benefit may point in the same direction.

### The Principle of Non-maleficence

The principle of non-maleficence requires that healthcare providers do not intentionally harm their patients, either through acts of commission or omission (Beauchamp and Childress, 2013, 150). If introducing responsibility-based policies inflicts harm on patients, this clash with the non-maleficence principle makes it reasonable to object to such policies. There are several ways these policies can create harm. Again, the more severe the illness, the more hesitant we might be to introduce measures of lower priority. For bad outcomes, concerns of harshness may arise (Anderson, 1999). If a policy of lower priority inflicts costs on those who are responsible, we should be hesitant toward asking them for footing such a large bill (or a significant fraction of it). One reason would be that we will impose a further disadvantage on someone who is already badly off in terms of being ill or at high risk of becoming so (Cavallero, 2011). Also, adding such costs may also affect behavior. Expectations about higher expenses for treatment or insurance may hinder people from seeking needed health care or insurance in the first place (but could perhaps also act as a deterrent toward risky behavior). Furthermore, if contagious diseases are untreated, they can be a health risk to others. Thus, policies based on responsibility can end up imposing harm on people lacking the ability to adequately protect themselves from the risk. On the other hand, there is also a sense in which responsibility-sensitive measures avoid certain forms of harm associated with non-responsibility-sensitive policies. When those who are responsible for their illness are treated for free, the costs are passed on to others.

Another issue potentially creating harm arises in the process of assessing whether a person is responsible for health-related actions. It could be that clarifying this is demeaning or intrusive to the person under assessment (both to those who can be considered responsible and those who cannot) (Wolff, 1998; Anderson, 1999). It could thus be the case that even though we could come to know whether a person is responsible in the sense established by the listed criteria, the process of revealing this would be of such a character that it possesses a threat to the trust ideally structuring a health personnel–patient relationship.

### The Principle of Justice

The principle of justice requires that we distribute goods and services, including medical goods, and services, fairly (Beauchamp and Childress, 2009). The question of distributive justice rests on the fact that some goods and services are in short supply, and thus some fair mechanism of allocating scarce resources must be determined.

Those stressing the inclusion of responsibility measures in health care do so precisely because they believe it to be fair. To illustrate, luck egalitarians (e.g., Cohen, 1989; Arneson, 2000) believe that people should be held responsible for the consequences of free and informed choices. In a healthcare setting, for example, this could imply that a person who, due to avoidable, and risky choices, contracts a disease should be given lower priority. Of course, those who disagree that justice has to do with personal responsibility will deny that justice requires such an allocation.

Furthermore, even for those who agree with the general idea of holding people responsible for the consequences of their choices, it can be problematic to determine when and to what extent patients should be held responsible for their healthcare-related choices. To illustrate, if health behavior is prevalent in specific socioeconomic groups, measures giving lower priority to those who succumb to disease as a result of that behavior can be problematic. If the existing distribution of benefits and burdens in society is to some extent unfair, then giving lower priority to those responsible for their health disadvantages may unfairly exacerbate such inequalities (Cavallero, 2011). Thus, the introduction of such measures should not only be based on an awareness of the extent to which socioeconomic inequalities affect people’s health behaviors and thus undermine the extent to which people control their health behavior; it should also take into account whether these socioeconomic inequalities are inherently fair (Albertsen and Knight, 2015). Especially for measures introducing co-payments for treatment, the existence of socioeconomic inequalities may be deemed problematic.

### Structural Features of Actions: Second Filtering

Again, we can set up the litmus test: To what conditions included in responsibility catering policies could the adversely affected reasonably object, and following from that filtration, what conditions are then left to potentially base policies on? From the discussion above, we can say that being met by risk-sharing policies only seems fair if one is considered responsible for the action (according to the conceptualizing conditions of the first filtering process), the policy would not be a disincentive to health-promoting behavior, and the risk for causing a healthcare need can be justified as considerable. What kinds of actions would satisfy these criteria? It seems to us that these conditions match mainly with hazardous activities that do not clearly promote health and that involve the risk of negative health consequences in terms of injuries, illness (not contagious), or death if they go wrong.

For a responsibility-based policy introduced at the bedside, the acceptable conditions for holding someone responsible become more fine-grained, and challenging. In such circumstances, the decision-makers must be able to establish a convincing causal relation between certain actions, and the unhealthy condition, as well as confirming the high probability that the act(s) in question contributed to the condition. On the assumption that the causality requirement is met, we are still left with a number of further concerns, that is, *the uncertainty related to: clarifying responsibility in terms of the presence of autonomous choices, the severity of the disease, the obtainable beneficial treatment, the cost of treatment, and the potential harm to others associated with non-treatment, as would be the case for contagious diseases*. However, from the discussion above, we can sum up some conditions that do not strongly conflict with the bioethical principles and therefore allow for the transition from being responsible to being held responsible. Such conditions can then be seen as constituting the basis for responsibility-sensitive measures at the point of care: (1) the responsibility for the action causing the need is not contested by anyone subjected to the policy, (2) the harm inflicted on those considered responsible by the policy is small, (3) there is little potential to benefit from a treatment, and (4) the monetary requirements are minimal. In cases like this, the objections to introducing responsibility-based policy can be outweighed by the reasons to introduce the policies in the first place, namely, to save money for the treatment of others. However, there are reasons to question whether there are any kinds of healthcare needs that correspond to this set of conditions. Moreover, it is not obvious that much money would be saved by policies based on the cluster of these conditions. The administrative costs associated with implementing responsibility catering policies might possibly even outweigh the resources saved.

### A Framework for Assessing Responsibility as a Criterion for the Distribution of Health Care

**Fig 1.**
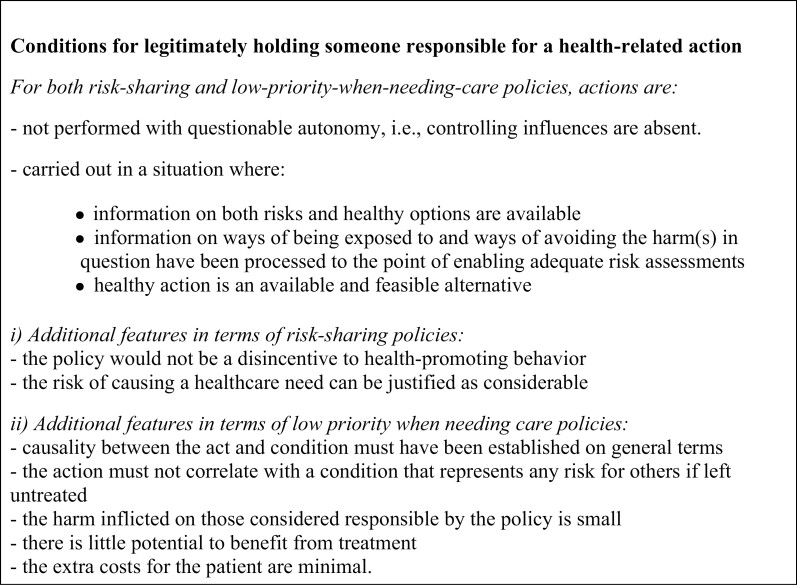
A framework of structural features of action-healthcare need-pairings for assessing responsibility as a criterion for the distribution of health care.

We can now summarize the structural features of actions and potentially corresponding healthcare needs on which it can be fair to base responsibility-sensitive measures in figure 1. The clusters of criteria presented in this framework can be used as part of an analytical tool for revising practice or developing new, justified policies.

## V. CONCLUSION

We have argued that fair and legitimate policies developed to hold people responsible for their health-related actions through sanctions should meet certain generic conditions. By first filtering such conditions through a conceptual analysis of being responsible, and then against the core values of public health encouragements, and the four ethical principles structuring Western healthcare services, we isolated a set of generic structures of actions for which it could be reasonable to hold people responsible (without lapsing into moral judgments about who deserves what). This framework can serve as an analytical tool for decision-makers, prevent unjustified practices, and encourage the design of legitimate health policies.

## Footnotes

1 Allocating responsibility can be left to the discretion of healthcare personnel to implement on a case-by-case basis, or it can be institutionalized in terms of standardized policies addressing groups of individuals according to specified characteristics. Whether to allow for individualized assessments or not is an interesting discussion in its own right. However, we set this discussion aside, since we are interested in the narrower focus on standardized policies in this paper, which minimizes the room for professional discretion.

2 Note that by doing so, we are not claiming that fairness considerations are what actually drive real-world politics in this area. As the efficiency rationale can also be used in support of benefitting individuals to make the least-risky choice, we briefly touch on this in the discussion of reasons for holding people responsible.

3 Following Watson (1996), it has become increasingly common to distinguish between different kinds of responsibility. In this paper, we focus on responsibility as *accountability.* For discussion, see Scanlon (1998), Shoemaker (2015), Smith (2015), and Carlsson (2019) draws a distinction between responsibility as attributability and substantive responsibility. The former notion concerns whether a person is a proper subject to praise or blame for what she has done. The latter concerns whether the person can reasonably object to the burdens and obligations she has to bear as a result of her actions.

4 See, for example, Rosen (2004) and Zimmerman (2008). This is, however, not the only way of understanding the relation between responsibility and blameworthiness. According to Fischer and Tognazzini (2011, 18–22), for instance, an agent can be morally responsible for a morally wrong action *without* being blameworthy for that action. To be morally responsible, according to them, only requires (a) that the agent is “a sensible target of the reactive attitudes,” and (b) that the agent’s action is morally wrong. However, they argue that (a) and (b) do not entail *blameworthiness*. After all, an agent can be a sensible target of the reactive attitudes and do something morally wrong, while still having a valid excuse for that action. According to Fischer and Tognazzini, it is an *open question* whether an agent who is morally responsible for some morally wrong action is also blameworthy for that action. See also Scanlon’s distinction between responsibility as attributability and substantive responsibility mentioned above.

5 In the literature, there are several different ways of specifying what appropriateness should mean, including fairness (Wallace, 1994), desert (McKenna, 2012; Pereboom, 2014; Carlsson, 2017), and fittingness (Graham, 2014; Shoemaker, 2015).

6 Moreover, sometimes we lack the standing to blame. If I just behaved rudely, it might be hypocritical for me to blame you for being rude.

7 We note, however, the thin line between making moral, and non-moral assessments of responsibility and underscore the importance of not letting these perspectives merge in discussions about responsibility in health.

8 In their influential work on this topic, John Martin Fischer and Mark Ravizza (1998) make a distinction between regulative control and guidance control. Regulative control requires the ability to act otherwise in actual circumstances. Guidance control does not require the availability of alternative possibilities in the actual circumstance, but it requires that the agent’s actions proceed from a mechanism that is “moderately reason responsive” and that this mechanism is the agent’s own. These notions of control are of great importance to the debate about free will, but we are not concerned with them in this paper.

9 We sometimes have a certain amount of *indirect* volitional control over our reasons-responsive attitudes: we can increase the likelihood of coming to a certain belief by thinking or reading, or experiencing a sad emotion by vividly imagining a sad situation.

10 Other philosophers use different terms when expressing a similar notion of control. McHugh (2017) calls it attitudinal control, whereas Hieronymi (2008) refers to it as evaluative control.

11
Rosen (2004), Levy (2005), and Zimmerman (2008) argue that volitional control is necessary for moral responsibility. Hieronymi (2004), Smith (2005), and Portmore (2019) argue that rational control is sufficient.

12 Note that the notion of fair opportunity does not presuppose an incompatibilist notion of control. Brink and Nelkin (2013) take the notion to be compatibilist. They argue that a fair opportunity to avoid wrongdoing requires that the agent could have acted otherwise, but they go on to give a compatibilist reading of “could have acted otherwise.”

13 For discussion of this tracing strategy, see Vargas (2005), Fischer and Tognazzini (2009), and Portmore (2019).

14 Leichter argues that given that we now know more about the potential dangers of smoking, it has become more reasonable to consider people responsible for smoking (Leichter, 2003).

## References

[CIT0001] Albertsen, A. 2015. Tough luck and tough choices: Applying luck egalitarianism to oral health. Journal of Medicine and Philosophy 40(3):342–62. 25870307 10.1093/jmp/jhv001

[CIT0002] ———. 2016. Drinking in the last chance saloon: Luck egalitarianism, alcohol consumption, and the organ transplant waiting list. Medicine, Health Care, and Philosophy 19(2):325–38. 26838765 10.1007/s11019-016-9684-7

[CIT0003] ———. 2020. Personal responsibility in health and health care: Luck egalitarianism as a plausible and flexible approach to health. Political Research Quarterly 73(3):583–95.

[CIT0004] Albertsen, A., and C. Knight. 2015. A framework for luck egalitarianism in health and healthcare. Journal of Medical Ethics 41(2):165–9. 24505116 10.1136/medethics-2013-101666

[CIT0005] Andersen, M. M. 2014. What does society owe me if I am responsible for being worse off? Journal of Applied Philosophy 31(3):271–86.

[CIT0006] Andersen, M. M., S. O. Dalton, J. Lynch, C. Johansen, and N. Holtug. 2013. Social inequality in health, responsibility and egalitarian justice. Journal of Public Health 35(1):4–8. 23436200 10.1093/pubmed/fdt012

[CIT0007] Andersen, M. M., and M. E. J. Nielsen. 2016. Personal responsibility and lifestyle diseases. Journal of Medicine and Philosophy 41(5):480–99. 27473408 10.1093/jmp/jhw015

[CIT0008] Anderson, E. S. 1999. What is the point of equality? Ethics 109(2):287–337.

[CIT0009] Arneson, R. J. 2000. Luck egalitarianism and prioritarianism. Ethics 110(2):339–49.

[CIT0010] Bærøe, K. 2008. Priority setting in health care: On the relation between reasonable choices on the micro-level and the macro-level. Theoretical Medicine and Bioethics 29(2):87–102. 18528781 10.1007/s11017-008-9063-3

[CIT0011] Bærøe, K., and R. Baltussen. 2014. Legitimate healthcare limit setting in a real-world setting: Integrating accountability for reasonableness and multi-criteria decision analysis. Public Health Ethics 7(2):98–111.

[CIT0012] Bærøe, K., and C. Cappelen. 2015. Phase-dependent justification: The role of personal responsibility in fair healthcare. Journal of Medical Ethics 41(10):836–40. 26269464 10.1136/medethics-2014-102645

[CIT0013] Beauchamp, T. L., and J. F. Childress. 2009. Principles of Biomedical Ethics. 6th ed. New York: Oxford University Press.

[CIT0014] ———. 2013. Principles of Biomedical Ethics. 7th ed. Oxford, United Kingdom: Oxford University Press.

[CIT0015] Brink, D. O. and D. K. Nelkin. 2013. Fairness and the architecture of responsibility. In Oxford Studies in Agency and Responsibility, ed. David Shoemaker, 284–313. Oxford, United Kingdom: Oxford University Press.

[CIT0016] Brown, A. 2005. If we value individual responsibility, which policies should we favour? Journal of Applied Philosophy 22(1):23–44.

[CIT0017] Brown, R. C. H. 2013. Moral responsibility for (un)healthy behaviour. Journal of Medical Ethics 39(11):695–8. 23315854 10.1136/medethics-2012-100774 PMC3812898

[CIT0018] Brown, R. C. H., H. Maslen, and J. Savulescu. 2019. Responsibility, prudence and health promotion. Journal of Public Health 41(3):561–65. 30007299 10.1093/pubmed/fdy113 PMC6785701

[CIT0019] Buyx, A. M. 2008. Personal responsibility for health as a rationing criterion: Why we don’t like it and why maybe we should. Journal of Medical Ethics 34(12):871–4. 19043112 10.1136/jme.2007.024059

[CIT0020] Cappelen, A. W., and O. F. Norheim. 2005. Responsibility in health care: A liberal egalitarian approach. Journal of Medical Ethics 31(8):476–80. 16076974 10.1136/jme.2004.010421 PMC1734208

[CIT0021] ———. 2006. Responsibility, fairness and rationing in health care. Health Policy 76(3):312–9. 16112248 10.1016/j.healthpol.2005.06.013

[CIT0022] Carlsson, A. B. 2017. Blameworthiness as deserved guilt. The Journal of Ethics 21(1):89–115.

[CIT0023] ———. 2019. Shame and attributability. In: Oxford Studies in Agency and Responsibility vol. 6, ed. D. Shoemaker, 112–39. Oxford: Oxford University Press.

[CIT0024] Cavallero, E. 2011. Health, luck and moral fallacies of the second best. The Journal of Ethics 15(4):387–403.

[CIT0025] Clarke, R. 2014. Omissions: Agency, Metaphysics, and Responsibility. New York: Oxford University Press.

[CIT0026] Cohen, G. A. 1989. On the currency of egalitarian justice. Ethics 99(4):906–44.

[CIT0027] Daniels, N., and J. E. Sabin. 2002. Setting Limits Fairly: Can we Learn to Share Medical Resources? Oxford: Oxford University Press.

[CIT0028] Dworkin, G. 1981. Taking risks, assessing responsibility. Hastings Center Report 11(5):26–31. 7309497

[CIT0029] Feiring, E. 2008. Lifestyle, responsibility and justice. Journal of Medical Ethics 34(1):33–6. 18156519 10.1136/jme.2006.019067

[CIT0030] Fischer, J. M., and M. Ravizza. 1998. Responsibility and Control: A Theory of Moral Responsibility. Cambridge: Cambridge University Press.

[CIT0031] Fischer, J. M., and N. A. Tognazzini. 2009. The truth about tracing. Noûs 43(3):531–56.

[CIT0032] ———. 2011. The physiognomy of responsibility. Philosophy and Phenomenological Research 82(2):381–417.

[CIT0033] Friesen, P. 2016. Personal responsibility within health policy: Unethical and ineffective. Journal of Medical Ethics 44(1):53–8. 27660291 10.1136/medethics-2016-103478

[CIT0034] GBD Causes of Death Collaborators. 2017. Global, regional, and national age-sex specific mortality for 264 causes of death, 1980–2016: A systematic analysis for the Global Burden of Disease Study 2016. Lancet 390(10100):1151–210. 28919116 10.1016/S0140-6736(17)32152-9 PMC5605883

[CIT0035] Graham, P. A. 2014. A sketch of a theory of moral blameworthiness. Philosophy and Phenomenological Research 88(2):388–409.

[CIT0036] Heidenreich, P. A., J. G. Trogdon, O. A. Khavjou, J. Butler, K. Dracup, M. D. Ezekowitz, E. A. Finkelstein, et al. 2011. Forecasting the future of cardiovascular disease in the United States. A Policy Statement from the American Heart Association 123(8):933–44. 10.1161/CIR.0b013e31820a55f5 21262990

[CIT0037] Hieronymi, P. 2004. The force and fairness of blame. Philosophical Perspectives 18(1):115–48.

[CIT0038] ———. 2008. Responsibility for believing. Synthese 161(April):357–73.

[CIT0039] Kapiriri, L., O. F. Norheim, and D. K. Martin. 2007. Priority setting at the micro-, meso- and macro-levels in Canada, Norway and Uganda. Health Policy 82(1):78–94. 17034898 10.1016/j.healthpol.2006.09.001

[CIT0040] Le Grand, J. 2013. Individual responsibility, health, and health care. In: Inequalities in Health: Concepts, Measures, and Ethics, eds. N. Eyal, S. Hurst, O. F. Norheim, and D. Wikler. Oxford: Oxford University Press.

[CIT0041] Leichter, H. M. 2003. “Evil habits” and “personal choices”: Assigning responsibility for health in the 20th century. Milbank Quarterly 81(4):603–26. 14678481 10.1046/j.0887-378X.2003.00296.x PMC2690243

[CIT0042] Levy, N. 2005. The good, the bad and the blameworthy. Journal of Ethics and Social Philosophy 1(2):1–16.

[CIT0043] ———. 2014. Consciousness and Moral Responsibility. New York: Oxford University Press.

[CIT0044] ———. 2019. Taking responsibility for responsibility. Public Health Ethics 12(2):103–13. 31384300 10.1093/phe/phz001 PMC6655467

[CIT0045] Maluka, S., P. Kamuzora, M. San Sebastián, J. Byskov, B. Ndawi, and A. -K. Hurtig. 2010. Improving district level health planning and priority setting in Tanzania through implementing accountability for reasonableness framework: Perceptions of stakeholders. BMC Health Services Research 10(1):322. 21122123 10.1186/1472-6963-10-322 PMC3009977

[CIT0046] McHugh, C. 2017. Attitudinal control. Synthese 194(8):2745–62.

[CIT0047] McKenna, M. 2012. Conversation and Responsibility. New York: Oxford University Press.

[CIT0048] Moore, M. S., and H. M. Hurd. 2011. Punishing the awkward, the stupid, the weak, and the selfish: The culpability of negligence. Criminal Law *and Philosophy* 5(2):147–98.

[CIT0049] Nielsen, L., and D. V. Axelsen. 2012. Three strikes out: Objections to Shlomi Segall’s luck egalitarian justice in health. Ethical Perspectives 19(2):307–16.

[CIT0050] Pereboom, D. 2014. Free Will, Agency, and Meaning in Life. New York: Oxford University Press.

[CIT0051] Portmore. D. 2019. Opting for the Best. New York: Oxford University Press

[CIT0052] Rakowski, E. 1993. Equal Justice. Oxford: Clarendon.

[CIT0053] Rawls, J. 1993. Political Liberalism. New York: Columbia Press.

[CIT0054] Reiser, S. J. 1985. Responsibility for personal health: A historical perspective. The Journal of Medicine and Philosophy 10(1):7–17. 3884725 10.1093/jmp/10.1.7

[CIT0055] Rosen, G. 2004. Skepticism about moral responsibility. Philosophical Perspectives 18(1):295–313.

[CIT0056] Sandman, L., E. Gustavsson, and C. Munthe. 2016. Individual responsibility as ground for priority setting in shared decision-making. Journal of Medical Ethics 42(10):653–8. 27495235 10.1136/medethics-2015-103285

[CIT0057] Scanlon, T. M. 1998. What we Owe to Each Other. Cambridge, MA: Harvard University Press.

[CIT0058] Schmidt, H. 2008. Bonuses as incentives and rewards for health responsibility: A good thing? Journal of Medicine and Philosophy 33(3):198–220. 18567903 10.1093/jmp/jhn007

[CIT0059] ———. 2009. Just health responsibility. Journal of Medical Ethics 35(1):21–6. 19103938 10.1136/jme.2008.024315

[CIT0060] Segall, S. 2010. Health, Luck, and Justice. New Jersey: Princeton University Press.

[CIT0061] Sher, G. 2009. Who Knew?: Responsibility Without Awareness. New York: Oxford University Press.

[CIT0062] Shoemaker, D. 2015. Responsibility from the Margins. Oxford: Oxford University Press.

[CIT0063] Smith, A. M. 2005. Responsibility for attitudes: Activity and passivity in mental life. Ethics 115(2):236–71.

[CIT0064] ———. 2015. Responsibility as answerability. Inquiry 58(2):99–126.

[CIT0065] Strawson, P. 2005. Freedom and resentment. In Free Will: Concepts and Challenges, vol. 1, ed. J. M. Fischer, 37–57. New York: Routledge.

[CIT0066] Thornton, V. 2009. Who gets the liver transplant? The use of responsibility as the tie breaker. Journal of Medical Ethics 35(12):739–42. 19948929 10.1136/jme.2009.029967

[CIT0067] Traina, G., P. E. Martinussen, and E. Feiring. 2019. Being healthy, being sick, being responsible: Attitudes towards responsibility for health in a public healthcare system. Public Health Ethics 12(2):145–57. 31384303 10.1093/phe/phz009 PMC6655377

[CIT0068] Vargas, M. 2005. The trouble with tracing. Midwest Studies in Philosophy 29(1):460–75.

[CIT0069] Veatch, R. M. 1980. Voluntary risks to health: The ethical issues. Journal of the American Medical Association 243(1):50–5. 6765977

[CIT0070] Veatch, R. M. and P. Steinfels. 1974. Case studies in bioethics: Who should pay for smokers’ medical care? The Hastings Center Report 4(5):8–10. 4426746

[CIT0071] Wallace, R. J. 1994. Responsibility and the Moral Sentiments. Cambridge, Massachusetts: Harvard University Press.

[CIT0072] Watson, G. 1996. Two faces of responsibility. Philosophical Topics 24(2):227–48.

[CIT0073] Wolff, J. 1998. Fairness, respect, and the egalitarian ethos. Philosophy and Public Affairs 27(2):97–122.

[CIT0074] Zimmerman, M. J. 2008. Living with Uncertainty: The Moral Significance of Ignorance. (Cambridge Studies in Philosophy). Cambridge: Cambridge University Press.

